# When Ears Drive Hands: The Influence of Contact Sound on Reaching to Grasp

**DOI:** 10.1371/journal.pone.0012240

**Published:** 2010-08-17

**Authors:** Umberto Castiello, Bruno L. Giordano, Chiara Begliomini, Caterina Ansuini, Massimo Grassi

**Affiliations:** 1 Dipartimento di Psicologia Generale, Università di Padova, Padova, Italy; 2 Schulic School of Music, McGill University, Montreal, Quebec, Canada; University College London, United Kingdom

## Abstract

**Background:**

Most research on the roles of auditory information and its interaction with vision has focused on perceptual performance. Little is known on the effects of sound cues on visually-guided hand movements.

**Methodology/Principal Findings:**

We recorded the sound produced by the fingers upon contact as participants grasped stimulus objects which were covered with different materials. Then, in a further session the pre-recorded contact sounds were delivered to participants via headphones before or following the initiation of reach-to-grasp movements towards the stimulus objects. Reach-to-grasp movement kinematics were measured under the following conditions: (i) congruent, in which the presented contact sound and the contact sound elicited by the to-be-grasped stimulus corresponded; (ii) incongruent, in which the presented contact sound was different to that generated by the stimulus upon contact; (iii) control, in which a synthetic sound, not associated with a real event, was presented. Facilitation effects were found for congruent trials; interference effects were found for incongruent trials. In a second experiment, the upper and the lower parts of the stimulus were covered with different materials. The presented sound was always congruent with the material covering either the upper or the lower half of the stimulus. Participants consistently placed their fingers on the half of the stimulus that corresponded to the presented contact sound.

**Conclusions/Significance:**

Altogether these findings offer a substantial contribution to the current debate about the type of object representations elicited by auditory stimuli and on the multisensory nature of the sensorimotor transformations underlying action.

## Introduction

Reaching and grasping movements are among the most common actions we perform in our everyday lives. To perform these actions, different sensory modalities are used in concert to perceive and interact with multimodally specified objects and events. For example, crossmodal links between haptic information and visuomotor control, when reaching to grasp a visual target, have been reported in published experiments [1]–[3]. Participants in these experiments reached and grasped a visual target, a sphere of variable size, with one hand, while holding an unseen distractor, a sphere of a different size, in the other hand. When the target and the distractor differed in size, proprioceptively-guided manipulation of the distractor influenced the finger shaping of the visuomotor grasping of the target. Specifically, the amplitude of the maximum grip aperture (i.e., the maximum distance between the index finger and the thumb) was smaller, and the time to maximum grip aperture was earlier, when the distractor was smaller than the target, and vice versa.

Crossmodal action-perception effects have also been reported in studies that assessed the effects of olfactory information on visually guided reach-to-grasp movements [4]–[6]. Participants reached towards and grasped either a small or a large visual target in the absence or in the presence of an odor evoking either a small or a large object. When the ‘smell size’ was incongruent with the visual size, interference effects emerged in the kinematics of hand shaping. Specifically, when participants grasped a small target (e.g., a strawberry) in the presence of a ‘large’ odor (e.g., an apple) finger extension was greater than when no smell accompanied the reach-to-grasp movement. Similarly, when participants grasped a large target (e.g., an orange) in presence of a ‘small’ odor, a flexion pattern emerged that was not evident in absence of olfactory information. Together, these findings were taken as evidence that proprioceptive and olfactory information can influence the planning and control of reach-to-grasp movements.

While multisensory processes underlying reach-to-grasp movements have been reported, as detailed above, the potential role of auditory information has largely been neglected. Research on auditory cognition has revealed, for example, that untrained listeners are capable of correctly recovering a large number of properties of sound-generating objects and events based on sound information alone. These properties include the identity of the sound source [7], the material of a struck object [8], and its size [9]–[11].

Interestingly, accurate source perception has been documented not only when we hear the sounds generated by the actions of another person (e.g., perception of the relative position of clapping hands [12]; perception of the gender and posture of a walker [13], [14]), but also for sounds produced by our own motor activity (e.g., perception of the texture of a surface inspected with a rigid probe or with the fingers [15], [16]). Furthermore, neurophysiological and neuroimaging studies support the idea that this auditory information may be involved at the level of action representation. For example, it has been observed that neurons within the premotor cortex (area F5) of monkeys discharge when a monkey performs a specific manual action, and they also discharge when the monkey hears a sound that corresponds to the action [17], [18]. Importantly, some of these neurons required both visual and auditory input to accompany the action event. These audiovisual neurons discharge during the execution of specific motor actions, suggesting that they are part of the hand-action vocabulary that has been described within the ventral premotor cortex [19]. Recently, Gazzola and colleagues [20] found evidence of activation within the ventral premotor cortex in humans during both motor execution and listening to the sound of an action made by the same effector.

In the present study, we investigated whether an action sound, generated by the interaction between the fingers and a grasped object, alters the kinematics of reaching and grasping for a visually presented stimulus. Our motivation was to establish whether auditory information can influence action representations, and to assess at which level it may occur.

To investigate these issues we considered contact-point events; that is, when the fingers make contact with a grasped stimulus. These events give rise to salient sensory signals in the auditory and the visual modalities, together with signals in the tactile modality. For example, the eyes are usually directed to the stimulus to determine possible contact points, and the interaction between the fingers and the surface of the grasped object generates a sound signal. Thus, contact cues potentially provide an opportunity for sensorimotor integration and intermodal alignment. This integration and alignment may help in the derivation of multimodal sensorimotor correlations that in turn support the planning and generation of purposeful motor commands [21]. It is well established that the brain can automatically integrate temporally correlated information occurring in the somatosensory, auditory, and visual modalities, and neural activity common to all three stimulus modalities is present in the parietal and frontal cortices [e.g., 22], and in the posterior superior temporal sulcus [23].

## Experiment 1

The aim of Experiment 1 was to assess the effect of sound information on the dynamics of a reach-to-grasp movement to a visual object. We recorded the sound produced by the fingers when they make contact with objects covered with different materials (i.e., aluminum, paper, string, wool), and we presented one of the recorded contact sounds to participants at different times before and after the participants initiated a reach towards a visual object. The presented contact sound either corresponded to (i.e., congruent condition) or differed from (i.e., incongruent condition) the sound generated by contact with the visual object. A control condition using a synthetic sound was also included.

We anticipated that the auditory information would affect kinematics differently depending on the congruency between the delivered contact sound and that elicited by the visual stimulus at contact. We expected that in the incongruent conditions (e.g., the delivery of a ‘string’ sound and the presentation of a visual target covered by paper) interference at the level of intermodal integration would emerge. The mismatch between two sensory modalities signalling different information regarding the same event might, for example, result in an increase in the time to initiate and perform the action, together with a delayed occurrence of key kinematic landmarks. Conversely, in the congruent conditions, in which both the auditory and the visual information are characterized by a similar contact sound (e.g., the delivery of a ‘string’ sound and the presentation of a visual target covered by string) we expected facilitatory effects due to an optimal link between two modalities signalling the same contact event. In this condition the time to initiate and perform the action should be shorter, and key kinematic landmarks should be anticipated.

### Materials and Methods

#### Ethics statement

The experimental procedures were approved by the Institutional Review Board at the University of Padua, and were in accordance with the Declaration of Helsinki (Sixth revision, 2008). All participants gave their informed written consent to participate in the study.

#### Participants

Twenty right-handed participants (10 females and 10 males, mean age 25.6 years) took part in the experiment. All participants reported normal hearing and normal, or corrected to normal, vision. Handedness was determined by using the Oldfield [24] questionnaire. All subjects were naïve to the purpose of the experiment.

#### Visual stimuli

The stimuli were four plastic spheres of 8 cm diameter and a weight of 100 g. The stimuli were covered with different materials (i.e., aluminium, paper, string, wool). The stimuli were all colored red. Because we wanted to make sure that the stimuli differed solely on the basis of the evoked contact sound, we performed a pilot study in which participants (4 females and 4 males), with the same characteristics as those who took part in the main experiment, were asked to perform reach to grasp movements towards the visual stimuli (6 trials per material). Statistical details for this pilot testing are reported within the ‘data analysis’ section. We anticipate that no differences across materials were found.

#### Sound stimuli

The impact sound resulting from fingers making contact with objects covered by either aluminium, paper, string, or wool was recorded within an Industrial Acoustics Company double-walled soundproof booth. Participants performed a natural prehensile movement involving the opposition of the thumb with the other fingers. A microphone (Behringer ECM8000) was positioned 25 cm from the surface of the objects used as target stimuli. The sound signal captured by the microphone was delivered to a firewire audio interface (MOTU 828mkII; sampling rate = 44.1 kHz, resolution = 16 bit, duration 200 ms) and stored on the hard drive of a computer. The sound used for the ‘control’ condition was a synthetic complex tone derived from the first ten harmonics of a 780 Hz fundamental frequency (duration 200 ms). All harmonics had identical amplitude and phase.

#### Apparatus and procedure

**Figure 1:**
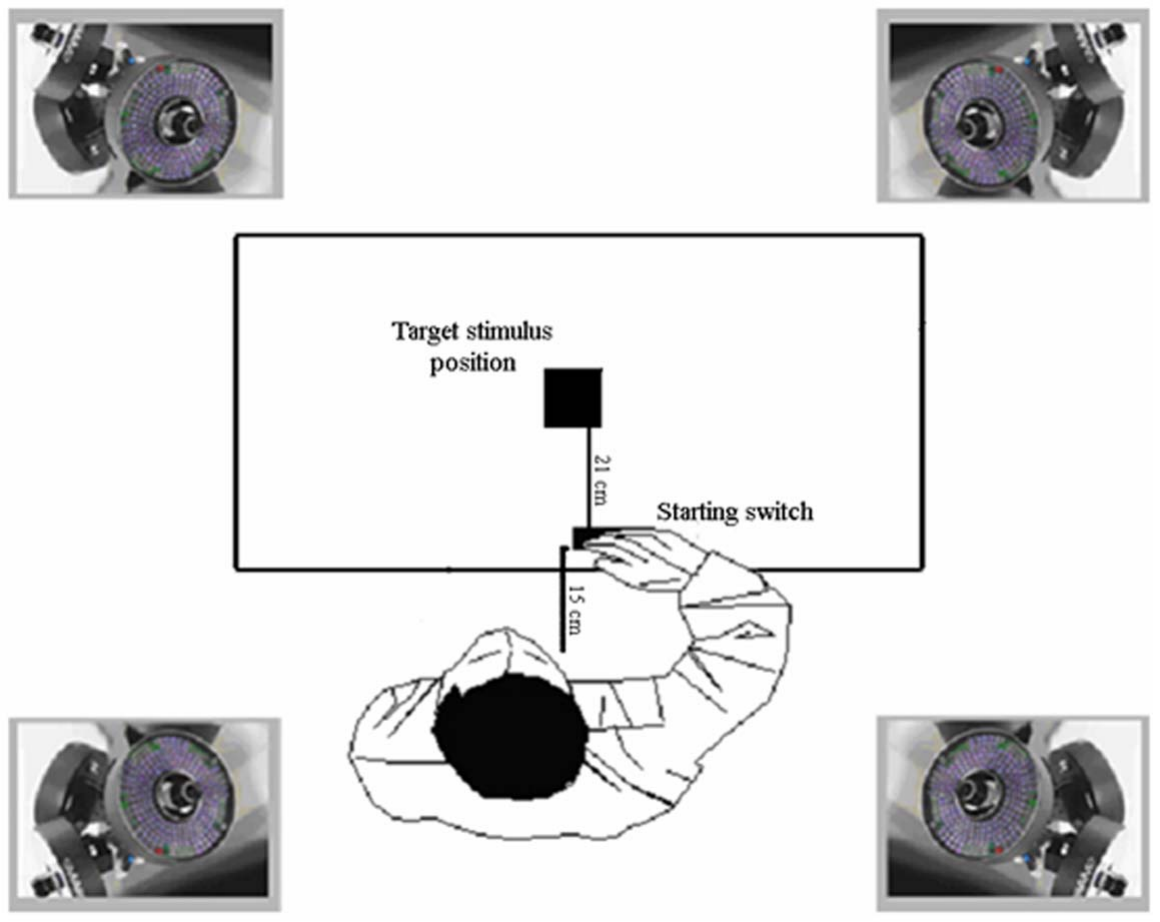
Experimental set-up. This figure depicts the location at which the four infrared cameras were positioned together with the participants' start position and the stimulus location.

The apparatus is illustrated in Figure 1. Prior to the beginning of each trial, the visual stimulus was placed at the centre of a 2 cm by 2 cm square. To control the onset of the visual stimulus and to prevent vision between trials, participants wore glasses with liquid-crystal shutters (Plato spectacles; Translucent Technologies). Under computer control, the shutters change from translucent to transparent within 10 ms and return to translucent in 2 ms. The participant was seated with the sagittal mid-line of the body aligned with the sphere. A start key was located 3 cm away from the edge of the table and 15 cm anterior to the participant's midline (see Fig. 1). The distance between the starting switch and the visual stimulus was 21 cm. While waiting for the start of each trial, each participant was instructed to maintain the ulnar side of the hand placed upon the starting pad, the shoulder slightly flexed, the forearm semi-pronated, the wrist extended (5°–10°), and a gentle opposition between the pads of the index finger and thumb.

Participants attended a practice session in which they performed a reach-to-grasp action towards the object covered with one of the four considered materials both in the presence or absence of the sounds previously recorded. The sound was presented by means of headphones (Sennheiser, HD 580, overall sound pressure level ∼30 dB SPL). In this session participants experienced grasping all materials and all possible sound/material combinations. No feedback regarding the relationship between sound and the material covering the object was given. Then they attended two experimental sessions (approximately 1 h duration each). In one session (off-line session), sound stimuli were presented at each of three different moments, 100 ms, 250 ms, or 500 ms, before object exposure. In another session (on-line session), sound stimuli were delivered 100 ms, 250 ms, or 500 ms after the initiation of the movement. The order of the two sessions was counterbalanced between participants. Each trial began when the spectacles worn by the participants became transparent, and thus allowed the participant to see the stimulus. Participants were asked to initiate a reach movement immediately after the stimulus became visible. Participants were not given specific instructions on how to grasp the stimulus, apart from being asked to grasp it firmly enough so that it could be lifted. Note that wearing the headphones prevented participants from hearing any environmental sound including the actual contact sound. The experimenter visually monitored each trial to ensure participants' compliance with these requirements. During the practice and the experimental sessions sounds were presented at the same overall intensity.

The pre-recorded sounds were classified as ‘congruent’ when the presented sound corresponded to the material covering the to-be-grasped stimulus, as ‘incongruent’ when the presented sound did not correspond to the material covering the to-be-grasped stimulus, and as ‘control’ where a synthetic sound was presented. For both the ‘off-line’ and the ‘on-line’ sessions, participants performed 24 trials for both the congruent and the control conditions (six trials for each type of material). For the incongruent-sound condition, participants performed two trials for each of the 12 possible combinations of the visual and auditory materials, for a total of 24 trials.

#### Kinematic recording

Movements were recorded using an ELITE motion analysis system (Bioengineering Technology & Systems [B|T|S]). Four infrared cameras (sampling rate 100 Hz), placed 120 cm away from each of the four corners of the table (see Fig. 1), captured the movement of infrared reflective markers (0.25-mm diameter) taped to the following points on the participants' right upper limb: (1) wrist–dorsodistal aspect of the radial styloid process; (2) thumb–ulnar side of the nail; and (3) index finger – radial side of the nail. A fourth marker was attached to the top of the stimulus. Markers were fastened using double-sided tape. Co-ordinates of the markers were reconstructed with an accuracy of 0.2 mm over the field of view. The standard deviation of the reconstruction error was 0.2 mm for the vertical (Y) axis and 0.3 mm for the two horizontal (X and Z) axes.

#### Data analysis

Motion recordings were initially filtered using a linear, finite impulse-response, high-pass filter (cutoff frequency, 10 Hz). The reaching component was calculated from the spatial trajectory and the tangential speed of the marker on the wrist. The grasp component was computed based on the distance between the markers located on the index finger and on the thumb (i.e., grip aperture), along with the spatial trajectory of the fingers.

Reaction time (RT) was defined as the time interval between clearing of the crystal liquid lenses and the release of the start key on which the hand was resting. Movement duration (MD) was calculated as the time between the release of the start key and the time at which the index finger and the thumb closed on the object and remained stationary for at least two frames (20 ms). For the reaching component we calculated the time at which maximum peak velocity (TPV) was reached, and the deceleration time (DT: the time from maximum peak velocity to the end of the movement). For the grasp component we considered the time at which maximum grip aperture was reached (TGA), and closing time (CT: the time from when maximum grip aperture was reached – TGA – to the end of grasp). An ANOVA with sound delivery time (off-line vs on-line), interval (150 ms, 250 ms, 500 ms) and type of sound (congruent, incongruent, and control), as within-subjects factors, was performed. Data were checked for normality, and univariate and multivariate outliers, with no serious violations noted. Results from the ANOVAs were explored with post-hoc contrasts. In the case of multiple tests, the probability returned by each test was adjusted with the Bonferroni correction (alpha level = 0.05) for the number of tests.

For the pilot testing an analysis of variance (ANOVA) with type of material (aluminium, paper, string, wool) as a within-subjects factor for each of the considered dependent measures was performed. No significant differences across materials were detected (RT: F(3,21)  = 1.04, *p* = 0.74; MD: F(3,21)  = 1.22, *p* = 0.31, TPV: F(3,21)  = 1.17, *p* = 0.22; DT: F(3,21)  = 1.08, *p* = 0.43; TGA: F(3,21 = 2.03, *p* = 0.67; CT: F(3,21)  = 1.34, *p* = 0.53).

### Results

**Figure 2:**
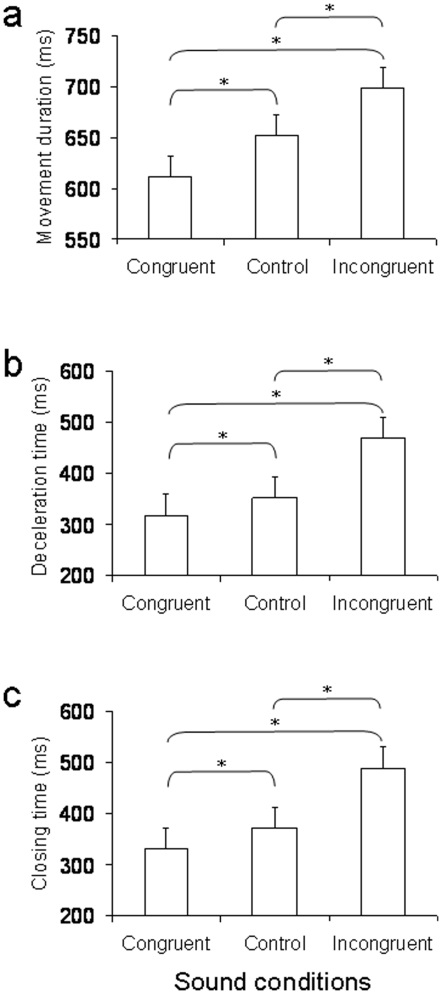
Kinematic results for Experiment 1. Average movement duration (panel ‘a’), deceleration time (panel ‘b’) and closing time (panel ‘c’) for the congruent, control and incongruent sound conditions. Error bars represent standard error of means. Asterisks indicate significant differences.

**Figure 3:**
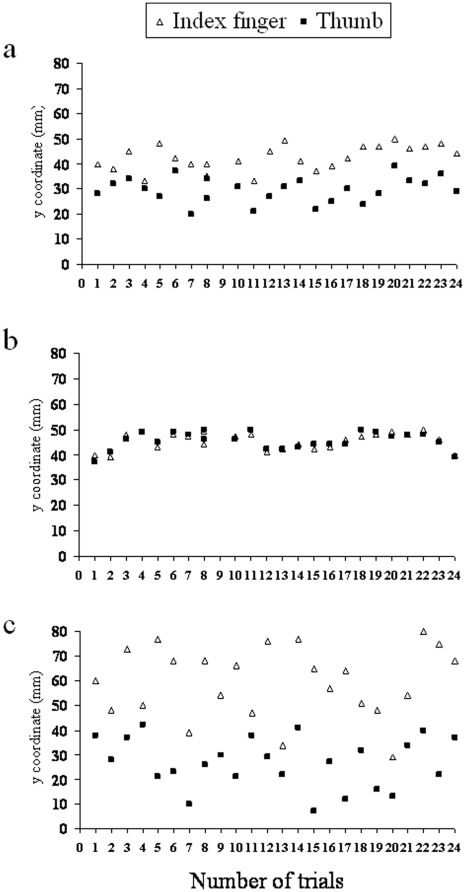
Contact points variability for Experiment 1. Graphical representation of contact points variability for the index finger and thumb for the control (panel ‘a’), the congruent (panel ‘b’) and the incongruent (panel ‘c’) sound condition. Single trials for a representative participant (n. 14) are depicted.

No significant main effects were observed for the factors sound delivery time, interval, and type of sound for RT and kinematic variables associated with the first phase of the action (i.e., TPV, TGA; *p_s_*>0.05). Furthermore, no significant interaction was found between these factors (*p_s_*>0.05). Thus, the timing and the nature of the presented sound did not affect action planning. However, the main factor, type of sound, was significant for MD [*F*(2, 38)  = 31.28; *p*<.0001], DT [*F*(2, 38)  = 15.02; *p*<.0001], and CT [*F*(2, 38)  = 21.17; *p*<.0001]. The nature of the presented sound affected measures related to the homing phase of the action. The effect of the type of sound was independent of the time at which the sound was delivered, as shown by the lack of a significant interaction between type of sound and sound delivery time (*p*>0.05). Post-hoc tests revealed that MD, DT, and CT were shorter for the ‘congruent’ than for the ‘control’ condition, and they were longer for the ‘incongruent’ than for the ‘control’ condition (Figure 2a–c; *p_s_*<.05), regardless of the point in time at which the sound was delivered. Because the effects reported above were all related to the final sequences of the action, we decided to look at possible effects of experimental manipulation on the variability of the contact point for the index finger and the thumb. Variability was calculated on the basis of the trigonometric relationship between the *y* axis position at which these two fingers contacted the stimulus and the marker placed on the top of the stimulus. As shown in Figure 3 for a representative participant, variability for the index finger and thumb contact points was much higher for the incongruent than for the control condition, and was lower for the congruent than for the control condition.

### Discussion

The present results indicate that the performance of a reach-to-grasp movement was influenced by the level of congruency between the presented contact sound and the actual contact sound usually experienced by participants upon touching the visual stimulus. The prolongation of DT and CT, together with a higher contact point variability for the ‘incongruent’ condition relative to the ‘control’ condition, is suggestive of interference effects, whereas the detection of shorter DT and CT, together with a smaller contact-point variability when comparing the ‘congruent’ with the ‘control’ condition, is suggestive of facilitation effects. Together, these findings indicate that the contact information elicited by the sound is incorporated on-line within the motor plan developed for reach to the visual target. Facilitation effects may be explained as an on-line integration of the two modalities for the same event, which, in turn, leads to a faster and more coherent action. As an alternative to the multisensory integration account a priming account might also explain the reported facilitation effects, at least for the condition in which the sound is delivered before movement initiation. In this view, the motor plan elicited by the sound may prime the motor plan established for the visual target. In other words, when a preceding sound elicits a motor plan which is congruent with the motor plan subsequently established for the visual target, then facilitation emerges. This view opens up the idea that other means of priming object material such as saying the name of the material or showing a picture of it might be sufficient to modify reaching behaviour. This is a possibility which should be tested in future research.

One might suggest that the facilitation effects reported here for the congruent condition result from the fact that participants experienced more sound/material pairings for the congruent than for the incongruent combinations. Participants may have become more familiar with congruent than incongruent material/sound combinations. To rule out this possible familiarity effect, an analysis of variance (ANOVA) considering trial order for the congruent condition (1 vs 6) as within-subjects factor was performed. No effect was found for any of the dependent measures that were considered (*p_s_*>0.05).

Interference effects may be explained as a result of an increased response uncertainty that affects transport of the hand towards the target, and affects how the fingers are placed on the target. These effects occurred independently of the time at which the sound was presented.

An important aspect of the present findings is that participants may have delayed consideration of the contact sound to the point at which, during a reach-to-grasp action, the contact sound should have effect. This delay may explain why the dependent measures specifically concerned with the execution of the end phase of the grasping action (i.e., CT, DT, and contact points variability) appear to be modulated by the nature of the sound, whereas those concerned with movement planning (i.e., RT) and the first stage of the movement were not (e.g., TPV), regardless of when during the reach-to-grasp timeline the contact sound was presented. For example, TPV, which occurs roughly at the 30% point of total movement duration [25], [26], may reflect planning more than control. Further support for the idea that the sound effect is deferred until the very end of the action comes from the finding of a lack of modulation of the TGA, a size-dependent parameter that occurs at roughly the 70% point of movement total duration [25], [26]. Effects observed near the end of a reach-to-grasp movement cannot be thought of as necessarily reflecting control processes alone. For example, it has been proposed that movement duration may reflect processes that occur before movement initiation [27]. Indeed, we found a decrease in movement duration for the congruent-sound condition, and an increase in movement duration for the incongruent-sound condition. However, the fact that in the present experiment movement duration may reflect control more than planning is supported by the evidence that longer movements tend to result almost entirely from an increase in the amount of time spent in deceleration, as has been demonstrated in published studies [e.g. 28–30].

## Experiment 2

The aim of Experiment 2 was to assess the effect of sound information on finger contact points. To this purpose, we presented a sound that was always congruent with the material covering either the upper of the lower half of the stimulus, before and after movement initiation. The crucial measure was in which of these two halves of the stimulus the index finger and thumb contacted the object. If the ‘sound’ effect observed in Experiment 1 has the ability to ‘pilot’ fingers contact points, then the sound should systematically influence towards which half of the object the fingers are positioned.

### Materials and Methods

#### Participants

Twenty right-handed participants (10 females and 10 males, mean age 25 years), with the same characteristics as those participating in Experiment 1, took part in the experiment. None of the participants had participated in Experiment 1.

#### Visual and sound stimuli

The size and weight of the stimuli were similar to the sizes and weights of stimuli used in Experiment 1. However, the upper and lower halves of the stimulus were each covered with a different material (e.g., paper/wool).

#### Apparatus, procedures and kinematic recording

The apparatus was almost identical to that used in Experiment 1. Participants conducted the preliminary kinematic assessment task (to control for any object-material effect), and the experimental task, as in Experiment 1. In the experimental phase, the sound delivered at different intervals before or after movement onset was always congruent with the material covering either the upper half or the lower half of the object. The range and the distribution of the sound delivery time was the same as for Experiment 1. Each material combination was administered in a counterbalanced and randomized order, for a total of 24 trials. The same sound was presented six times. No instructions were given to participants as to where the fingers should be located in order to lift the object.

#### Data analysis

The crucial measure was in which half of the stimulus object the index finger and thumb contacted the object. This measure was calculated on the basis of the end trajectories of the fingers with respect to a reference marker placed on the top of the stimulus. When grasping the object, participants could put both the index finger and thumb on one material, both digits on the other material, or one digit on one material and the other digit on the other material. Participants' grasps were thus classified as sound-congruent (i.e., both index finger and thumb touching the material congruent with the sound) and sound-incongruent (i.e., both index and thumb finger on the material incongruent with the sound, or the index finger on one material and the thumb on the other material). A binomial test was used to analyze the loci of participants' grasp.

### Results

**Figure 4:**
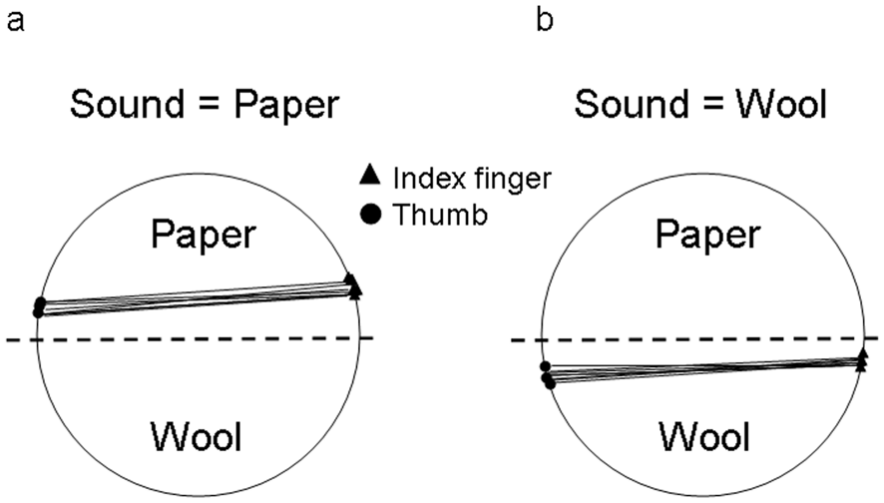
Graphical representation of contact points for the index finger and thumb in Experiment 2. The considered measure was in which half of the stimulus object the index finger and thumb contacted the object. This measure was calculated on the basis of the end trajectories of the fingers. A representative example of sound congruent grasp for the wool/paper material combination is presented (participant n. 9). The dashed line indicates the stimulus midline.

The binomial test revealed that participants used, almost exclusively, the sound-congruent grasp (84% of grasps; *z* = 4.77, *p*<.0001). For example, as shown in Figure 4, if the sound was related to ‘wool’, then fingers contacted the object on the half covered by wool. If the sound was related to ‘paper’, then the fingers contacted the object on the half covered by paper.

### Discussion

These findings suggest that the information carried by the contact sound may be as effective as the centre of mass in driving contact events [31]. It is noticeable that the sound-congruent grasp dominated, despite the inefficient positioning of the fingers that results from this grasp (i.e., both below or above the grip axis), which may have caused the object to roll along its horizontal axis (Figure 4a). This result is even more striking since a pre-requisite for the successful grasping of a spherical object is that grasping should occur along the midline axis of whatever axis passes through its centre [32]; that is, at the point at which tangential and gravity forces are essentially zero. It is worth noting, however, that in some circumstances it may be possible to predict the consequences of off-axis grasping by using anticipatory mechanisms. For example, in one study participants were constrained to grasp objects at points progressively further from the centre of mass [33]. It was demonstrated that participants used estimates of centre of mass based on visually available information about object geometry to perform a stable grasp by increasing grip force in proportion to, and in anticipation of, an increase in torque. It may be that our participants put in place similar anticipatory strategies, based on auditory and/or visual information, so as to anticipate and prevent a possible rolling of the object. Another possibility lies in the fact that the material covering the objects in all cases provided a level of friction which might have allowed for a ‘safe’ grasp even when the contact points did not optimize grasp stability.

## General Discussion

We investigated whether contact sound information contributes to representations that guide a reach-to-grasp movement. To this end, we adopted an approach that has already been successful at revealing the integration of multiple sensory modalities during the execution of visually guided grasping actions. That is, the presentation of task-irrelevant information in a different modality to that of task-relevant information. Results from Experiment 1 revealed that presenting a contact sound that is related to a material similar to that covering the visual target facilitated action execution. By contrast, presenting a sound associated with a material differing from that covering the visual target resulted in interference effects. Experiment 2 demonstrated the effect of sound information on the location of finger–stimulus contact.

The strength and the novelty of the present findings come chiefly from the observation that auditory information is not only indicative of the nature of sound-source events [34], but it is also indicative of information related to motor output [35]. Support for this contention comes from neurophysiological and behavioural evidence.

Neurophysiological data show that activity of neurons within motor areas can be driven by different types of sensory stimuli [35]. Of particular interest is the demonstration of polymodal neurons in the premotor cortex. For example, Graziano and colleagues [36] showed that some neurons within premotor area F4 can also be activated by auditory stimuli and their activity is also modulated by the intrinsic features (e.g., intensity) of the auditory stimulus. Importantly, the receptive fields of these neurons are in register with visual peripersonal receptive fields. Receptive fields for these neurons may be concerned with the various types of movements that are normally made inside this space, such as reaching and grasping. The natural conclusion is that auditory, as well as visual inputs are instrumental for providing sensory information for the different types of action represented at a premotor level. This conclusion has been further corroborated by the observation of neurons in the premotor cortex (area F5) of monkeys that discharge when a monkey makes a specific hand action also discharge when she hears the corresponding sound (e.g., breaking of a peanut; [17]). Thus, these neurons represent actions whether they are performed or only heard, and the neurons could be used to plan and execute actions.

Recent behavioural studies point to the benefits to motor performance of adding arbitrary, object unrelated, contact-sound cues when reaching to grasp objects in virtual reality environments [37], [38]. Here, we have extended this literature in two important ways. First, we provide further evidence that audio-motor interactions are likely to occur in humans, and that they extend to real world settings. Second, it is not merely the presence of contact sounds that alerts the motor system: the level of congruence between the auditory and the visual event appears to be an important determinant for the emergence of facilitation effects. If auditory cues simply raised the general level of alertness, we should have seen faster movement times whenever auditory cues were provided. However, when the visual and the auditory events do not correspond, interference effects emerge.

An informative aspect of the present results is the lack of RT effects. The sound manipulation did not produce any measurable effect at the level of movement preparation. Indeed, one would have expected that the increased response uncertainty dictated by the incongruent sound should have led to longer RTs for the initiation of the movement towards the target. Rather, all the reported effects occurred in measures related to movement execution.

An influential model of action posits that planning and on-line control each serve a specialized purpose different from the other, and each utilize distinct representations [27]. By this view, to fulfill its aims, planning must take into account a wide variety of spatial visual information, such as size, shape, and orientation of the target, together with other non-spatial characteristics of the target, including fragility, material, and weight. The control system, on the other hand, appears to be limited to the spatial characteristics of the target, allowing the added benefit of monitoring and occasionally adjusting motor programs in flight.

On the basis of this model, therefore, it would reasonable to assume that contact-sound information is under the control of the planning system. This is because contact sound might depend on object properties such as fragility, texture, and weight [27]. For instance, fingers might be positioned upon a fragile object more delicately than upon a plastic object, thus producing a different sound. But, as explained above, the reported effects were all concerned with kinematic measures indicating an involvement of the control system. A result which is in line with recent findings suggesting that people are able to adjust their programmed lifting forces online to a visually indicated change in the non-spatial variable weight [39].

Having said that, we cannot exclude the possibility that the reported effects rule out involvement of the planning system. It has been proposed that the two stages of action, namely planning and control, may be temporally overlapping [40–42]. Prior to movement initiation, planning is entirely responsible for the initial determination of all movement parameters. As movements progress, however, the influence of control on action increases. This gradual crossover between planning and control would have the benefit of allowing for smooth rather than jerky corrections [42]. Thus, it might well be that although contact sound is considered at the planning level, full consideration of this variable is given at the time it becomes task relevant (i.e., just before object contact).

In conclusion, to date there is sparse evidence from research with humans for the role of auditory information for the planning and execution of visually guided reach-to-grasp movements. Our results provide new insights to the perception of natural sounds and their use in the planning of actions. Furthermore the fact that auditory information affects grasping kinematics also when vision is present says something about the harmony between the organization of movement and multimodal stimuli. In this respect the present findings fuel the notion that multisensory integration is intimately involved in the production of movement.
